# Molecular targets of pomegranate (*Punica granatum*) in preventing cancer metastasis

**DOI:** 10.22038/ijbms.2019.34653.8217

**Published:** 2019-09

**Authors:** Naghmeh Ahmadiankia

**Affiliations:** 1 Cancer Prevention Research Center, Shahroud University of Medical Sciences, Shahroud, Iran

**Keywords:** Anoikis, Cell adhesion, Chemotaxis, Cytoskeleton dynamics, Extracellular matrix, Metastasis, Phytochemicals Pomegranate

## Abstract

Metastasis is the primary cause of mortality and morbidity among cancer patients and accounts for about 90% of cancer deaths. The most common types of treatment for cancer metastasis are chemotherapy and radiotherapy. However, such therapy has many serious side effects that could diminish the quality of life in patients. There is increased appreciation by the scientific community that natural compounds can be potential weapons in fighting against cancer. Interestingly, much evidence shows that pomegranate (*Punica granatum*) has great potential to inhibit tumor growth and metastasis. In this review, we discussed the molecular targets of pomegranate, specifically, those that are prerequisite for cancer metastasis. The search was performed in Google Scholar, Medline, Scopus, and PubMed using keywords such as metastasis, pomegranate, and signaling pathways. Some of the most important papers from the search results were included. Based on recent studies, some molecules, including those involved in cell-cell and cell-extracellular matrix adhesions, are affected by pomegranate. The other targets of pomegranate are modulators of cytoskeleton dynamics and regulators of cancer cell anoikis and chemotaxis. Furthermore, the antimetastatic effect of pomegranate may be attributed to molecular changes of the extracellular matrix. Pro-inflammatory and pro-angiogenic molecules are the other targets of pomegranate regarding cancer metastasis. A wide variety of molecules can be targeted by pomegranate to suppress tumor metastasis. A better understanding of the molecules regulated by pomegranate is needed to provide a rational basis for its clinical application.

## Introduction

Cancer is a major public health problem and one of the leading causes of death worldwide (1). It is estimated that metastasis is responsible for about 90% of cancer deaths (2). Cancer metastasis is a multistep process by which tumor cells spread from the primary tumor site to distant organs through blood and lymphatic vessels. The first step in cell dissemination is detachment from the primary tumor. Epithelial and endothelial cells will undergo apoptosis when detached from the extracellular matrix (ECM), a phenomenon referred to as anoikis; however, cancer cells develop anoikis resistance that is a critical step in metastasis. Anoikis resistance cells leave the primary tumor site, intravasate, circulate in vessels, and finally extravasate from the circulation in the distant organ where they form a secondary new tumor (3). During multiple steps of metastasis different molecules including those involved in cell–cell and cell–ECM adhesions, the modulators of cytoskeleton dynamics, the ones that organize ECM, and the signaling molecules involved in anoikis, chemotaxis, angiogenesis, and inflammatory responses are involved. Nowadays, chemotherapy as a conventional treatment is available for patients with cancer metastasis. However, the current chemotherapy agents fail to selectively and effectively kill cancer cells, and they usually have adverse side effects (4). Therefore, a safe, non-toxic, and highly effective regimen to fight cancer should be developed. Previous studies showed that consuming natural products is inversely associated with cancer incidence and mortality (5). Among them, pomegranate (*Punica granatum) *has been shown to inhibit the progression of cancer in different preclinical and clinical studies (6-8). It is an antioxidant-rich fruit containing different important bioactive compounds and alters many of the cellular and molecular processes involved in cancer progression (9). In this review article, we first discussed the phytochemical content of pomegranate and its chemotherapeutic properties. Then we explained studies on the molecular targets of pomegranate and its bioactive constituents regarding cancer metastasis. Understanding the mechanism of pomegranate action and identiﬁcation of its molecular targets provide a rational basis for its clinical application.

**Research method**

The current search was done in Google Scholar, Medline, Scopus, and PubMed through search words such as “anoikis, cell adhesion, chemotaxis, cytoskeleton dynamics, extracellular matrix, phytochemicals, metastasis, and pomegranate.” The search was carried out to include literature published as late as 1 September 2018.

**Phytochemical content of pomegranate**

Pomegranate is one of the oldest known fruit tree species, native to central Asian regions spreading from Iran to northern India, the Mediterranean area, and the Middle East. It is valued for its nutritional, medicinal, ornamental, and industrial properties (10). The pomegranate plant is divided into several parts, including fruit (peel, juice, and seeds), leaves, flowers, roots, and bark. There is a wide range of phytochemicals in its different parts, which are summarized in Table 1.

**Pharmacokinetics of pomegranate**

The most abundant polyphenols in pomegranate juice are ellagitannins that are hydrolyzable tannins releasing ellagic acid on hydrolysis (13). The absorption of ellagitannins is rather low in humans (14). Interestingly, gut microbiota metabolizes ellagitannins and ellagic acid leading to the formation of urolithin A, urolithin B, and isourolithin A (15). The metabolites of urolithin A and urolithin B are conjugated in the liver, then excreted in urine (16). Due to difference in gut microbiotica, different urolithin metabotypes (UMs) including UM-A, UM-B, and UM-0, are produced in some individuals (17). These urolithins circulate in the blood and reach many target organs where the effects of pomegranate ellagitannins are noted (18, 19). Previous studies showed that the highest production of urolithin was carried out in distal parts of the intestine in humans and pigs (20, 21). Urolithin appears in plasma and urine at significant concentrations, around 24 hr after ellagic acid intake (14, 22). Mertens-Talcott *et al*. investigated the absorption of a standardized extract from pomegranate in healthy human volunteers after the acute consumption of 800 mg of extract. Results indicated that ellagic acid from the extract is bioavailable, with an observed C(max) of 33 ng/ml at t(max) of 1 hr. They also identified the metabolites of urolithin A, urolithin B, hydroxyl-urolithin A, urolithin A-glucuronide, and dimethyl ellagic acid-glucuronide in plasma (23). Moreover, Seeram *et al*. demonstrated that consuming 180 ml of pomegranate juice concentrate was associated with maximum plasma concentrations of ellagitannins of 0.06 mmol/lit after 1 hr and the ellagitannins metabolites, total urolithin A of 0.14 mmol/lit, and total urolithinB of 0.01 at 6 hr (24). They also detected ellagic acid in human plasma at a maximum concentration (31.9 ng/ml) after 1 hr post ingestion, which was rapidly eliminated within 4 hr (25).

In another study, 1-liter pomegranate juice was given orally to healthy volunteers for five days. The results showed that punicalagin and ellagic acid present in the juice were not detected either in plasma or in urine however three microbial ellagitannin-derived metabolites were detected in both urine and plasma with great interindividual variability one day after pomegranate juice consumption (18). Interestingly, under simulated gastrointestinal conditions, up to 80% of the overall ellagic acid content was slowly released from fermented pomegranate wastes over 2 hr incubation at the slightly alkaline pHs simulating the small intestine environment (26). It was revealed that ellagic acid had poor absorption and rapid elimination after oral administration of pomegranate leaf extract, and part of it was absorbed from the stomach (27). In another study, pomegranate seed oil was given to the animals intragastrically for 40 days at concentrations of 1%, 2%, and 4%. The results have demonstrated that punicic acid from pomegranate seed oil was metabolized and incorporated in the form of conjugated linoleic acid in different rat tissues (28). Further research is warranted to determine the pharmacokinetics of pomegranate. 

**Clinical value of pomegranate**

Various chemical compounds in different parts of the pomegranate plant have crucial roles in the treatment of many diseases. For example, the rich phytochemical contents in seeds, leaves, and fruit have been attributed for their benefits in the treatment of diabetes (29, 30). Pomegranate extract has also shown protective effects against acute renal failure (31). Moreover, pomegranate seed has been reported to regulate urine discharge and control the burning sensation of urine (32). Punicalagin isolated from pomegranate peel exhibited anti-viral property against the herpes virus (33). Similarly, pomegranate polyphenol extract inhibits the influenza virus (34). Attenuation of atherosclerosis and lowered hypertension are the other clinical values of daily pomegranate juice intake (11). In traditional medicine, pomegranate peel extract could be beneficial in oxidative stress-induced degenerative diseases such as Alzheimer’s dementia (35). Additionally, the therapeutic role of peel and seed oil extract in preventing bone loss in preclinical models of osteoporosis has been proven (36, 37). Interestingly, pomegranate extract can accelerate the wound healing process in albino rats (38). Moreover, the beneficial effects of pomegranate peel have been proven in infertility treatment (39). Recently, a new flavone glucoside, together with ellagitannins and flavones extracted from flowers of pomegranate showed anti-obesity properties (40). Previous studies have revealed that pomegranate has anti-cancer effects as well. The most promising findings show that pomegranate extract impedes the progression of prostate cancer (41). Additional studies indicate that pomegranate may protect against bladder (42), breast (43), cervical (44), colon (45), leukemia (46), liver (47), lung (48), ovarian (49), pancreatic (50), and skin (51) cancers, as well. 

**Figure 1:**
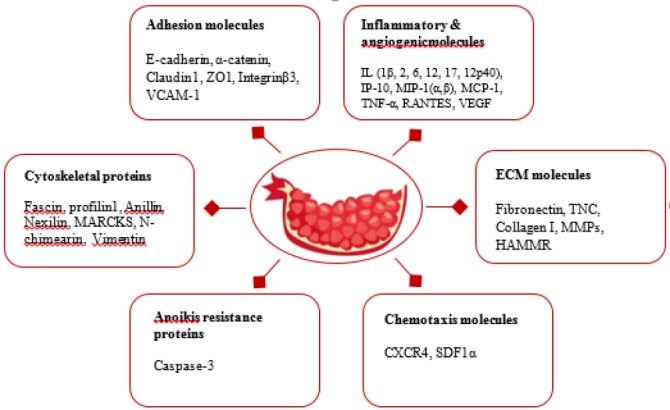
Schematic description of molecular targets of pomegranate with respect to cancer metastasis. CXCR4: C-X-C chemokine receptor type 4; HAMMR: Hyaluronic acid-mediated motility receptor/CD168; IL: Interleukin; IP-10: Induced protein-10; MARCKS: Myristoylated alanine-rich protein kinase C substrate; MCP: Monocyte chemoattractant protein; MIP-1: Macrophage inflammatory protein-1; MMP: Matrix metalloproteinase; RANTES: Regulated on activation, normal T Cell expressed and secreted; SDF1: Stromal cell-derived factor 1; TNC: Tenascin C; TNF-α: Tumor necrosis factorα; VCAM-1: Vascular cell adhesion molecule-1; VEGF: Vascular endothelial growth factor; ZO: Zonula occludens

In the next part, we bring together some of available *in vitro* and *in vivo* evidence relevant to the possible molecular targets of pomegranate with respect to cancer metastasis (Table 2 & Figure 1). 

**Molecular targets modulated by pomegranate regarding metastasis**

***Cell adhesion molecules and their regulators as targets of pomegranate***

**Figure 2:**
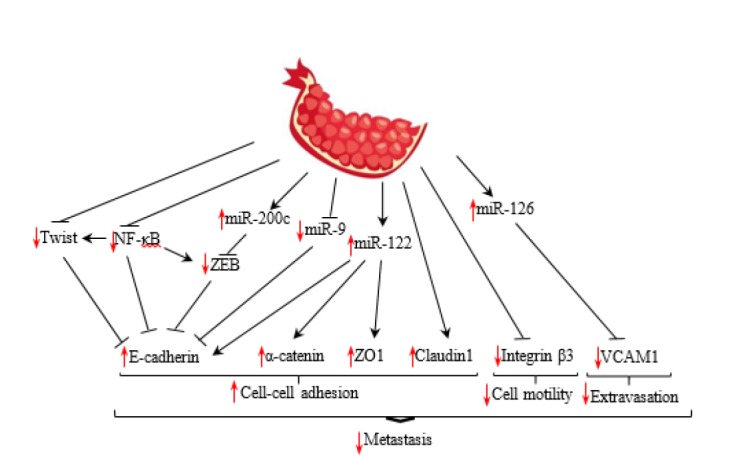
Schematic overview of adhesion molecules and their regulators as targets of pomegranate.NF-κB: Nuclear factor-κB; VCAM-1: Vascular cell adhesion molecule-1; ZEB1: Zinc finger E-box binding homeobox 1; ZO: Zonula occludens

Adhesion (attachment) and de-adhesion (detachment) are prerequisites for cellular motility and cancer metastasis (68). A group of cell adhesion molecules (CAMs) are involved in connecting cell to cell and cell to ECM components (69). A brief description of CAMs and their regulators, which are affected by pomegranate, is presented below (Figure 2).

**Figure 3:**
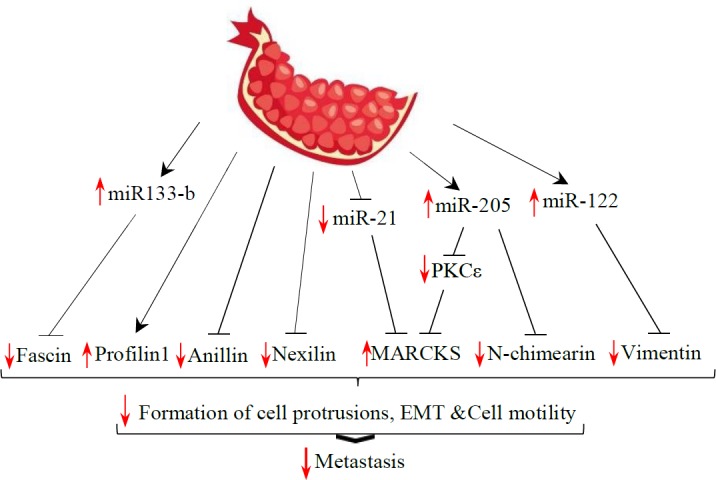
Schematic overview of cytoskeletal proteins and their regulators as targets of pomegranate. EMT: Epithelial mesenchymal transition; MARCKS: Myristoylated alanine-rich protein kinase C substrate; PKC: Protein kinase C

**Figure 4:**
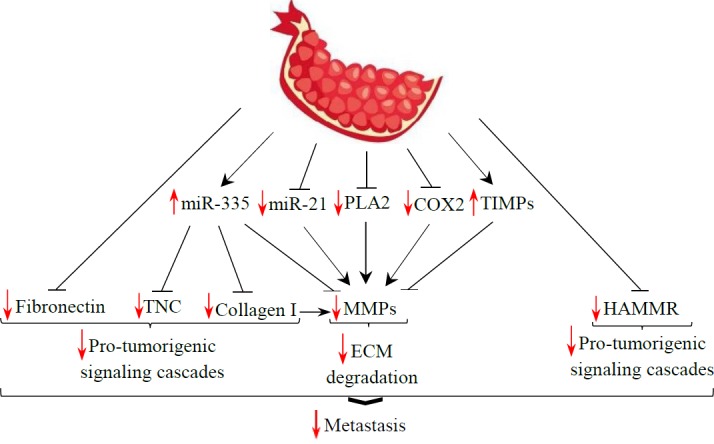
Schematic description showing the structural molecules of the extracellular matrix (ECM) as targets of pomegranate. COX-2: Cyclooxygenase-2; HAMMR: Hyaluronic acid-mediated motility receptor/CD168; MMPs: Matrix metalloproteinases; PLA2: phospholipase A2; TIMPs: Tissue inhibitors of metalloproteinases; TNC: Tenascin C

**Table 1:** Different classes of phytochemicals identified in various parts of the pomegranate tree and fruit [11, 12]

**Classes**	**Phytochemicals (tissue source)**
Ellagitannins, Gallotannins, and derivatives	Brevifolin (Leaf), Brevifolin carboxylic acid (Leaf, Flower, Heartwood), Brevifolin carboxylic acid 10-monopotassium sulphate (Leaf), Castalagin (Stem bark), Casuariin (Stem bark), Casuarinin (Peel, Stem bark), Corilagin (Peel, Leaf), Isocorilagin (Flower), Hippomanin A (Flower), Gemin D (Flower), Diellagic acid rhamnosyl(1→4) glucopyranoside (Heartwood), 1,2-Di-O-galloyl-4,6-O-(S)-hexahydroxydiphenoyl β-D-glucopyranoside (Flower), Ellagic acid (Peel, Flower, Leaf), 3,3’-Di-O-methylellagic acid (Seed), 3,3’,4’-Tri-O-methylellagic acid (Seed), 3-O-Methylellagic acid (Heartwood), 4,4’-Di-O-methylellagic Acid (Heartwood), 3’-O-Methyl-3,4-methylenedioxy-ellagic acid (Heartwood), Eschweilenol C (Ellagic acid 4-O-α-L-rhamnopyranoside) (Heartwood), Ethyl brevifolincarboxylate (Flower), Eucalbanin B (Aril), Eucarpanin T1 (Aril), Pomegraniin A (Aril), Pomegraniin B (Aril), Gallagic acid (Peel), Gallic acid 3-O-β-D-(6’-O-galloyl)-glucopyranoside (Flower), 6-O-Galloyl-2,3-(S)-hexahydroxydiphenoyl-D-glucose (Stem bark, Juice), 5-Galloylpunicacortein D (Heartwood), 2-O-Galloylpunicalin (2-O-Galloyl-4,6-(S,S)-gallagyl-D-glucose) (Heartwood, Stem Bark), Granatin A (Fruit, Leaf), Granatin B (Fruit, Leaf), 2,3-(S)-Hexahydroxydiphenoyl-D-glucose (Stem bark, Juice), Lagerstannin B (Peel), Lagerstannin C (Juice), 3-O-Methylellagic acid 4-O-α-L-rhamnopyranoside (Heartwood), 3,4’-O-Dimethylellagic acid 4-O-α-L-rhamnopyranoside (Heartwood), Oenothein B (Aril), Pedunculagin I (Peel, Stem bark), Pedunculagin II (Juice), 1,2,3,4,6-Penta-O-galloyl-β-D-glucose (Leaf), 3,4,8,9,10-Pentahydroxydibenzo [b,d] pyran-6-one (Urolithin M-5) (Leaf), Phyllanthusiin E (Flower), Pomegranatate (Flower), Punicacortein A (Stem bark), Punicacortein B (Stem bark), Punicacortein C (Stem bark, Peel), Punicacortein D (Stem bark), Punicafolin (Leaf), Punicalagin A (Peel, Stem bark, Aril, Juice, Root), Punicalagin B (Peel, Stem bark, Aril, Juice, Root), Punicalin (Peel, Stem bark, Aril, Juice, Heartwood), Punicatannin A (Flower), Punicatannin B (Flower), Punigluconin (Stem bark), Strictinin [1-O-Galloyl-4,6-(S)-hexahydroxydiphenoyl-D-glucose] (Leaf), Tellimagrandin I (Peel), Tercatain [1,4-Di-O-galloyl-3,6-(R)-hexahydroxydiphenoyl-β-glucopyranose] (Leaf), Terminalin (Gallagyl dilactone) (Stem bark), 1,2,4,6-Tetra-O-galloyl-β-D-glucose (Leaf), 1,2,3-Tri-O-galloyl-β-glucopyranose (Leaf), 1,2,4-Tri-O-galloyl-β-glucopyranose (Leaf), 1,2,6-Tri-O-galloyl-β-glucopyranose (Leaf, Flower), 1,3,4-Tri-O-galloyl-β-glucopyranose (Leaf), 1,4,6-Tri-O-galloyl-β-glucopyranose (Leaf), 3,4,6-Tri-O-galloyl-β-glucopyranose (Flower), Valoneic acid dilactone (Juice, Peel)
Flavonoids and anthocyanins	Hovetrichoside C (Flower), Phloretin (Juice), Phlorizin (Flower), Eriodictyol-7-O-α-L-arabinofuranosyl (1-6)-β-D-glucoside (Stem bark), Granatumflavanyl xyloside (Flower), Naringin (Naringenin-7-O-rhamnoglucoside) (Peel), Naringenin-4′methyl ether 7-O-α-L-arabinofuranosyl(1-6)-β-D-glucoside (Stem bark), Pinocembrin (Juice), Punicaflavanol (Flower), Apigenin (Peel), Apigenin 4′-O-β-glucopyranoside (Leaf), Luteolin (Peel, Flower), Luteolin 3′-O-β-glucopyranoside (Leaf), Luteolin 4′-O-β-glucopyranoside (Leaf), Cynaroside (Luteolin 7-O-glycoside) (Peel), Luteolin 3′-O-β-xylopyranoside (Leaf), Tricetin (Leaf), Daidzein (Seed), Genistein (Seed), Amurensin (Noricaritin 7-β-D-glucopyranoside) (Juice), Kaempferol (Peel), Astragalin (Kaempferol 3-O-glucoside) (Peel), Kaempferol-3-O-rhamnoglucoside (Juice), Myricetin (Peel), Phellatin (Juice), Quercetin (Juice, Leaf, Seed, Peel), Hirsutrin (Quercetin-3-O-glucoside) (Peel), Quercimeritrin (Quercetin-7-O-glucoside) (Peel), Quercetin 3-O-rhamnoside (Peel), Rutin (Quercetin-3-O-rutinoside) (Juice), Quercetin-3,4′-dimethyl ether 7-O-α-L-arabinofuranosyl(1-6)-β-D-glucoside (Stem bark), Cyanidin (Juice), Chrysanthemin (Cyanidin-3-O-glucoside) (Juice), Cyanin (Cyanidin-3,5-di-O-glucoside) (Juice), Antirrhinin (Cyanidin-3-O-rutinoside) (Juice), Catechin-cyanidin-3-hexoside (Juice), Delphinidin (Juice), Myrtillin (Delphinidin-3-O-glucoside) (Juice), Delphinidin-3,5-di-O-glucoside (Juice), Pelargonidin (Juice), Callistephin (Pelargonidin-3-O-glucoside) (Juice), Pelargonin (Pelargonidin-3,5-di-O-glucoside) (Juice), Catechin (Peel, Juice, Leaf), Epicatechin (Peel, Juice, Leaf, Seed), Epicatechin gallate (Peel), Epigallocatechin-3-O-gallate (Fruit), Gallocatechin-(4→8)-catechin (Peel), Gallocatechin-(4→8)-gallocatechin (Peel), Catechin-(4→8)-gallocatechin (Peel), Procyanidin A2 (Peel), Procyanidin B1 (Peel), Procyanidin B2 (Peel), Procyanidin B3 (Peel)
Lignans	Conidendrin (Juice), Isohydroxymatairesinol (Peel), Isolariciresinol (Juice, Peel), Matairesinol (Wood knot), Medioresinol (Juice, Wood knot, Seed), Phylligenin (Peel), Pinoresinol (Juice), Secoisolariciresinol (Peel, Juice), Syringaresinol (Juice, Wood knot, Peel, Seed), Pomegralignan (Aril, Peel), Punicatannin C (Flower)
Triterpenoids and phytosterols	Asiatic acid (Flower), Betulinic acid (Betulic acid) (Leaf), Friedooleanan-3-one (Friedelin) (Stem and Root bark), Maslinic acid (Flower), Oleanolic acid (Flower), Punicanolic acid (Flower, Peel), Ursolic acid (Flower), Campesterol (Seed), Cholesterol (Seed), Daucosterol (Seed, Flower), β-Sitosterol (Seed, Flower), β-Sitosterol laurate (Peel), β-Sitosterol myristate (Peel), Stigmasterol (Seed)
Alkaloids and indolamines	N-(2’,5’-dihydroxyphenyl)pyridinium Chloride (Leaf), Hygrine (Root bark), Norhygrine (Root bark), Pelletierine (Stem and Root bark), *N*-methylpelletierine (Stem and Root bark), Norpseudopelletierine (Stem and Root bark), Pseudopelletierine (Stem and Root bark), 2-(2’-Hydroxypropyl)-∆^1^piperideine (Root bark), 2-(2’-Propenyl)-∆^1^piperideine (Root bark), Punigratane (2,5-Diheptyl-N-methyl pyrrolidine) (Peel), Sedridine (Root bark), Melatonin (Fruit extract), Serotonin (Fruit extract), Tryptamine (Fruit extract)
Fatty acids and lipids	Caproic acid (Hexanoic acid) (Juice), Caprylic acid (Octanoic acid) (Juice), Capric acid (Decanoic acid) (Juice), Lauric acid (Dodecanoic acid) (Seed), Myristic acid (Tetradecanoic acid) (Seed, Fruit), Myristoleic acid (9-cis-Tetradecanoic acid) (Seed), Palmitic acid (Hexadecanoic acid) (Seed, Fruit), Palmitoleic acid (Hexadec-9-enoic acid) (Seed, Fruit), Punicic acid (9Z, 11E, 13Z-octadecatrienoic acid) (Seed), Linoleic acid (cis, cis-9,12-Octadecadienoic acid) (Seed, Fruit), α-Linolenic acid (All-cis-9,12,15-octadecatrienoic acid) (Seed, Fruit), γ-Linolenic acid (All-cis-6,9,12-octadecatrienoic acid) (Seed, Fruit), Oleic acid (9Z-octadecenoic acid) (Seed, Fruit), Stearic acid (Octadecanoic acid) (Seed, Fruit), α-Eleostearic acid (9Z, 11E, 13E-octadecatrienoic acid) (Seed), β-Eleostearic acid (9E, 11E, 13E-octadecatrienoic acid) (Seed), Catalpic acid (9E, 11E, 13Z-octadecatrienoic acid) (Seed), Arachidic acid (Eicosanoic acid) (Seed, Fruit), Gadoleic acid (9Z-icosenoic acid) (Seed), Behenic acid (Docosanoic acid) (Seed), Nervonic acid (cis-15-Tetracosenoic acid) (Seed, Fruit), 1-O-9E,11Z,13E- Octadecatrienoyl glycerol (Seed, Peel), 1-O-Isopentyl-3-O-octadec-2-enoyl glycerol (Seed, Peel), Tri-O-punicylglycerol (Seed), Di-O-punicyl-O-octadeca-8Z, 11Z, 13E-trienylglycerol (Seed), N-palmitoyl cerebroside (Seed)
Organic acids and phenolic acids	Ascorbic acid (Leaf, Peel, Seed, Juice), Citric acid (Juice, Leaf, Peel, Seed), Fumaric acid (Juice), L-Malic acid (Juice, Leaf, Peel, Seed), Oxalic acid (Juice, Leaf, Peel, Seed), Quinic acid (Juice), Succinic acid (Juice, Leaf, Peel, Seed), Tartaric acid (Juice), Caffeic acid (Peel, Juice, Seed, Leaf), Chlorogenic acid (Juice), Cinnamic acid (Juice), o-Coumaric acid (Juice), p-Coumaric acid (Peel, Juice, Seed, Leaf), cis-p-Coumaric acid (Peel), Coutaric acid (Peel), 7,8-Dihydroxy-3-carboxymethylcoumarin-5-carboxylic acid (Flower), Ferulic acid (Juice, Seed, Peel, Leaf), Gallic acid (Peel, Juice, Flower), Methyl gallate (Heartwood), Neochlorogenic acid (5-O-Caffeoylquinic acid) (Peel, Juice), Protocatechuic acid (Peel, Juice), Vanillic acid (Peel, Juice), Coniferyl 9-O-[*b*-D-apiofuranosyl(1→6)]-O-*b*-D-glucopyranoside (Seed), Sinapyl 9-O-[*b*-D-apiofuranosyl(1→6)]-O-*b*-D-glucopyranoside (Seed)
Other compounds	Catechol (Juice), Coumestrol (Seed), Icariside D1 (Seed), Phenylethylrutinoside (Seed), Syringaldehyde (Juice)

**Table 2:** Antimetastatic effects of pomegranate determined in *in* *vitro* and *in vivo* pre-clinical studies

**Cancer type**	**Studied Model**	***In vitro*** **/** ***in vivo*** **/clinical**	**Extract/Phytochemical**	**Mechanism**	**Ref**
Bladder	T24	*In vitro*	Extract (juice)	Inactivated PTEN/AKT/mTORC1 pathway via profilin 1 up-regulation	[42]
Breast	MDA-MB-231	*In vitro*	Extract (peel)	Decreased expression of β-catenin & EMT markers	[52]
	MDA-MB-231	*In vitro*	Extract (peel)	Down-regulation of metastasis-related genes	[53]
	MDA-MB-231, MCF-7	*In vitro*	Extract (seed oil)	Reduced secretion of inflammatory cytokines	[54]
	MDA-MB-231, MCF-7	*In vitro*	Juice or a combination of luteolin+ellagic acid+punicic acid	Reduced expression of pro-inflammatory cytokines/chemokines, adhesion molecules, cytoskeletal & ECM proteins, &EMT markers	[55]
	MDA-MB-231, MCF-7, MCF-10A	*In vitro*	Extract (seed oil), Fermented juice	Reduced expression of VEGF, inhibited angiogenesis	[56]
Colon	HT-29, AOM-induced ACF rats	*In vitro, in vivo*	Juice	Targeting miR-126-regulated pathways which contribute in anti-inflammatory & anti-angiogenic mechanisms	[45]
	HT-29	*In vitro*	Juice, Tannin, Punicalagin	Abolish TNFα-induced AKT activation resulting modulation of inflammatory cell signaling	[57]
Liver	DENA induced-rat hepatocarcinoma	*In vivo*	Emulsion	Suppression of the inflammatory cascade through modulation of NF- κB signaling pathway	[47]
Lung	A549, H1299, LL/2	*In vitro*	Extract (leaves)	Reduction of MMP-2 & MMP-9 expression	[48]
	A549	*In vitro*	Galactomannan (PSP001) isolated from the fruit rind	Down-regulation of VEGF & MMPs	[58]
Ovarian	A2780, ES-2 in nude mice	*In vitro, in vivo*	Fruit juice, Ellagic acid, Luteolin	Decreased expression of MMP2 & MMP9	[49]
	A2780	*In vitro*	Punicalagin	Suppression of MMPs acitivities	[59]
Prostate	LNCaP, LAPC4, CL1, DU145, LAPC4 in SCID mice	*In vitro, in vivo*	Extract	NF-κB blockade	[60]
	LNCaP, HUVEC, LAPC4 in SCID mice	*In vitro, in vivo*	Extract	Decreased expression of HIF-1α &VEGF	**[** 61 **]**
	DU145, PC3, LNCaP	*In vitro*	Luteolin+Ellagic acid+ Punicic acid	Decreased expression of oncogenic miRNAs & inhibition of the CXCR4/SDF1α chemotaxis axis, changes in the expression of cell adhesion & cytoskeletal proteins	[62]
	PC-3, PLS10	*In vitro*	Ellagic acid	Decreased secretion of MMP-2, inhibited collagenase IV activity	[63]
	DU145, PC3, TRAMP-C1	*In vitro*	Extract (peel)	Down-regulation of MMPs	[64]
	PC3	*In vitro*	Ellagic acid, Caffeic acid, Luteolin, Punicic acid	-	[65]
	PC3	*In vitro*	Extracts (peel, juice, seeds)	Decreased expression of PLA2	[66]
Renal	ACHN, SN12C	*In vitro*	Extract (juice)	Inhibition of NF-kB and JNK pathways, consequently inhibition of EMT phenotype	[67]
Skin	A375, B16F10 in C57BL/6 mice	*In vitro, in vivo*	Galactomannan (PSP001) isolated from the fruit rind	Down-regulation of VEGF & MMPs	[58]

CXCR4: C-X-C chemokine receptor type 4; ECM: Extracellular matrix; EMT: Epithelial mesenchymal transition; ERK: Extracellular signal-regulated kinase; HIF-1: Hypoxia-inducible factor 1; JNK: Jun N-terminal kinases; MMP: Matrix metalloproteinase; miRNA: microRNA; mTOR: Mammalian target of rapamycin; NF-κB: Nuclear factor-κB; PLA2: Phospholipases A2; PTEN: Phosphatase and tensin homolog; SCID: Severe combined immunodeficient; SDF1: Stromal cell-derived factor 1; TNF-α: Tumor necrosis factor-α; VEGF: Vascular endothelial growth factor.

*Cadherins*

Cadherins are transmembrane glycoproteins that mediate homophilic cell–cell adhesion. They constitute the core structural component of adherence junctions. E-cadherin is a member of this superfamily that is found in epithelial cells. The catenin complex, including α-catenin, β-catenin, γ-catenin, and p120 catenin link the intracellular domain of E-cadherin to the cytoskeleton (69). Disruption of E-cadherin or its linker proteins is the hallmark of epithelial mesenchymal transition (EMT), which is the most important mechanism behind the initiation of cancer metastasis. During EMT the cellular expression of cell adhesion molecules decreases, resulting in the formation of spindle-shaped morphology. Loss cell–cell connection allows tumor cells to disseminate and eventually metastasize. Several transcription factors have been implicated in the regulation of EMT, including the Snail family zinc finger transcription factors (Snail1 and Snail2), the Twist family basic helix–loop–helix transcription factors (Twist1 and Twist2), and the zinc finger E-box binding homeobox proteins (ZEB1 and ZEB2). The genes encoding cadherins, claudins, integrins, mucins, occludin, and ZO1 are repressed via these transcription factors (70, 71). 

In an attempt to unravel the anticancer properties of pomegranate, the effects of specific pomegranate juice components on the metastatic potential of prostate cancer cells were examined. They found that luteolin, ellagic acid, and punicic acid as important components of pomegranate juice significantly decreased invasion in prostate cancer cells partly through increasing the E-cadherin protein level. Moreover, affymetrix microarray and real-time PCR analysis revealed that ellagic acid, luteolin, and punicic acid decrease the expression of Twist (62). Twist is a transcriptional repressor of E-cadherin expression in breast cancer cells that suppresses E-cadherin through the E-box elements on its promoter (71). 

Moreover, it was revealed that pomegranate up-regulates miR-200c and down-regulates the expression of ZEB1 (52, 62). The miRNA-200 family has been shown to inhibit the transcription factors of ZEB1 and ZEB2, which are transcriptional repressors of the E-cadherin gene (72). It was assumed that pomegranate inhibits ZEB1 through up-regulation of miR-200c, leading to increased expression of E-cadherin (62).

Moreover, previous studies suggest that miR-9 can serve as a potential cancer biomarker. It plays an important role in cancer metastasis by activating the β-catenin pathway and inducing EMT via directly targeting E- cadherin (73). Interestingly, as reported previously, pomegranate decreased the expression of miR-9 and β-catenin, inhibited EMT and up-regulated expression of E-cadherin. Additionally, the expression of miR-122 was increased following pomegranate treatment in prostate cancer cells (52, 62). Previous studies showed that expression of miR-122 was correlated with an increased expression of E-cadherin and the linker protein of α-catenin (74). This evidence may be relevant to the antimetastatic property of pomegranate.

Interestingly, it was revealed that the adhesion molecule of E-cadherin is regulated by the NF-κB pathway (75). NF-κB is a family of five master transcription factors (NF-κB1/p105, NF-κB2/p100, RelA/p65, RelB, and c-Rel) that can form various heterodimers or homodimers and bind to consensus DNA sequences at promoter regions of responsive genes. In unstimulated cells, NF-κB dimers are inactive because of association with IκB proteins that prevent the nuclear localization of NF-κB and DNA binding. Stimulation of cells, for example by cytokines, activates IκB kinase (IKK) and results in the phosphorylation of IκB at serine residues. Phosphorylated IκB is subjected to ubiquitination and proteasome-mediated degradation, which results in the translocation of NF-κB to the nucleus, where it functions as a transcription factor (76, 77). NF-κB is involved in the regulation of EMT genes in breast cancer cells (78). Previous studies demonstrated that inhibition of NF-κB results in down-regulation of Twist1, Snail2, and ZEB2 and significant increase of E-cadherin expression (79). Interestingly, pomegranate extract inhibits the NF-κB pathway and consequently, reverses the EMT phenotype (60, 67). Study showed that this effect of pomegranate is mediated through the inhibition of IkB/IKK interaction. Flavonoids, as the main components of pomegranate, are responsible for this effect (80).

***Claudins***

Claudins are a family of transmembrane proteins that, along with occludins, are the most important components of the tight junctions. They are linked to the filamentous cytoskeleton via scaffolding proteins named zonula occludens (ZO) (81, 82). It was demonstrated that pomegranate increases gene expression of claudin-1 as a member of the claudin family. Moreover, the expression of miR-122 was increased following pomegranate treatment in prostate cancer cells (62). Previous studies showed that expression of miR-122 was correlated with increased expression of occludin and ZO-1 (74).

***Integrins***

Integrins are the key proteins responsible for cell-ECM adhesion. They link the cytoskeleton to ECM molecules providing required forces for cell migration. Cell-ECM adhesion also activate signaling pathways essential for cell motile function. Integrins consist of α and β subunits. The integrin subunits α3, α5, α6, αv, β1, and β3 are considered as biomarkers of metastasis (69). A study showed that consumption of pomegranate peel extract in a mice model of osteoporosis, significantly decreased the expression of integrin β3 (36). Considering previous studies, there is not enough investigation on the effect of pomegranate on the expression of integrins involved in cell-ECM interactions.

***Endothelial CAM***

Cell adhesion molecules, such as vascular cell adhesion molecule-1 (VCAM-1), intercellular adhesion molecule-1 (ICAM-1), and endothelial leukocyte adhesion molecule-1 (E-selectin), are known to modulate cell-endothelium interactions. They mediate initial adhesion of cancer cells to activated endothelium, their rolling, extravasation, and finally, the establishment of metastatic lesions (83). It was shown previously that pomegranate juice increased the expression of miR-126, leading to reduction of VCAM-1 expression in Azoxymethane treated rats (45). Several mRNAs such as VCAM-1 have a complementary sequence within their 3′-untranslated region for miR-126 (84).

Clearly, there is a lack of studies addressing the effects of pomegranate on the adhesion molecules in cancer cells. 

***Regulators of cytoskeleton dynamics as targets of pomegranate***

The cytoskeleton is a highly dynamic network that controls the cellular structure and movement. In this part, the effects of pomegranate on the molecules involved in the modulation of cytoskeleton dynamics are discussed (Figure 3).

Interestingly, fascin, as a key regulator of cytoskeleton dynamics, is down-regulated by pomegranate (62). Fascin is an actin-crosslinking protein that is required for the formation of actin-based cellular protrusions. Functional studies showed that fascin expression is involved in cell locomotion of both normal and neoplastic cells (85). It was revealed that fascin-1 mRNA was up-regulated in accordance with miR-133b down-regulation (86). Interestingly, pomegranate up-regulates the expression of miR-133b, which is accompanied by fascin down-regulation (62).

Profilin 1 is another member of actin-binding proteins, which participates in dynamic turnover and reconstruction of the actin cytoskeleton (87). Interestingly, decreased migration of urinary bladder cancer cells treated with pomegranate juice coincided with increased expression of profilin 1 (42).

Anillin and nexilin are scaffold proteins implicated in the regulation of cytoskeleton structure and also cell migration (88, 89). Interestingly, the expression of anillin and nexilin were decreased following pomegranate treatment (62). Myristoylated alanine-rich protein kinase C substrate (MARCKS) is localized to the plasma membrane and is an actin filament crosslinking protein. Phosphorylation by protein kinase C (PKC) or binding to calcium-calmodulin inhibits its association with actin and with the plasma membrane, leading to cell migration or invasion. It has been revealed that exogenous overexpression of MARCKS remarkably promoted cell attachment (90, 91).

Moreover, recent studies using prostate cancer cells showed that miR-21 promotes cell invasion by directly targeting MARCKS (92). Interestingly, pomegranate decreased the expression of miR-21 and increased MARCKS in prostate cancer cells (41, 62). Reversely, pomegranate juice increased the expression of miR-205 (41). It has been shown that miR-205 suppresses cell invasion in prostate cancer by inhibiting PKCε and N-chimearin (CHN1) (93). N-chimearin induces the formation of the actin-based structures lamellipodia and filopodia (94).

Vimentin is a kind of intermediate filament and one of the core components of the cytoskeletal network. It induces changes in cell shape, motility, and adhesion during EMT (95). In early works, the expression of vimentin in cancer cells was shown to be associated with a high risk of metastasis and poor prognosis (96). Bagheri *et al*. demonstrated that the expression of vimentin decreased in MDA-MB-231 cells following treatment with pomegranate peel extract (53). Previous studies showed that increased expression of miR-122 was correlated with reduced expression of vimentin (74). Increased expression of miR-122 following cell treatment with pomegranate components (62) may be correlated with decreased expression of vimentin in prostate cancer cells.

Taken together these results provide additional evidence that the antimetastatic effect of pomegranate may at least in part be mediated via genes and proteins involved in cytoskeleton dynamics.

***Molecules associated with anoikis resistance as targets of pomegranate***

Cancer cells acquire anoikis resistance to survive after detachment from the primary sites and travel through the circulatory and lymphatic systems to disseminate throughout the body (97). Researchers showed that ellagic acid could induce anoikis in ovarian carcinoma cells via inducing caspase-3 mediated apoptosis by increasing the Bax/Bcl-2 ratio (98). Moreover, the expression of Cyclooxygenase-2 (COX-2) was suppressed by pomegranate polyphenols (99). COX-2 inhibits anoikis by activating various pathways in different cancer types (100, 101). More study is needed to determine if pomegranate truly inhibits anoikis resistance in cancer cells.

***Regulators of cell chemotaxis as targets of pomegranate***

Chemotaxis of tumor cells in the surrounding microenvironment is essential for cancer cell dissemination during tumor progression and metastasis (102). C-X-C chemokine receptor type 4 (CXCR4) and its ligand, stromal cell-derived factor 1 alpha (SDF1α) are known as important chemotactic proteins in cancer metastasis (103, 104). 

The expression of CXCR4 and SDF1α were decreased after treatment with luteolin, ellagic acid, and punicic acid as the important components of pomegranate juice. They also inhibited prostate cancer cell migration toward SDF1α. Interestingly, luteolin, ellagic acid, and punicic acid together decrease the expression of Gα_13_, PI3K, and p-AKT proteins involved in the signaling downstream of CXCR4 (105). Among different microRNAs, miR-1 suppresses the expression of CXCR4 and its ligand SDF1α. It was found that miR-1 was up-regulated by luteolin, ellagic acid, and punicic acid (62). Furthermore, pomegranate decreased the expression of hypoxia-inducible factor-1 (HIF-1) (61). HIF-1 has been found to be a critical transcription factor for the expression of CXCR4 in a variety of cells, including tumor cells (106, 107). More studies are necessary to better understand the effects of pomegranate on the regulators of cancer cells chemotaxis.

***Structural molecules of ECM and their regulators as targets of pomegranate***

Since ECM is the key component of the tumor microenvironment, we asked whether ECM components are affected by pomegranate. Based on previous studies, pomegranate can prevent metastasis by affecting structural components of ECM (Figure 4). 

One of the major structural proteins in ECM is collagen. There is mounting evidence of the role of collagen-dense ECM in promoting metastasis in different cancers (108, 109). Collagen-I increases pro-tumorigenic signaling cascades such as focal adhesion kinase (FAK), src family kinases (SFKs), and extracellular regulated kinase (ERK)1/2 in tumor cells (110). It was demonstrated that type I collagen, secreted by stromal fibroblasts, may augment the aggressive characteristics of breast cancer cells through induction of matrix metalloproteinase (MMP)-9 (111). The main group of enzymes responsible for protein degradation in ECM is MMPs. The degradation of ECM by different members of the MMP family removes the physical barriers for tumor cells and reveals the hidden sites in the ECM, where various cell receptors can be bound to. The ability to degrade ECM is a critical requirement for tumor invasion (112). Previous studies showed that pomegranate or its ingredients such as ellagic acid, caffeic acid, luteolin, and quercetin decreased the expression of collagen 1 and/or MMP-1,-2,-9, and -13(48, 49, 53, 55, 62-64, 113, 114). This evidence suggests that the anti-metastatic role of pomegranate might be attributed, at least in part, to the reduction of collagen 1 and MMPs production by cancer cells.

Moreover, the level of phospholipase A2 (PLA2) transcript was decreased by polyphenols of pomegranate fermented juice, peel, and seed oil (62). PLA2 is a cytosolic enzyme responsible for prostaglandin production. It was shown that inhibition of prostaglandin reduced the release of MMPs and inhibited invasion in prostate cancer cells (115). Additionally, the expression and activation of MMPs are directly correlated to the expression of COX-2 in tumor cells (116). Interestingly, polyphenols in pomegranate decreased the expression of COX-2 (117), which may lead to down-regulation of MMPs and inhibition of metastasis.

Tissue inhibitors of metalloproteinases (TIMPs) are inhibitors of active MMPs (118). Previous studies revealed that pomegranate extract or its constituents up-regulate the expression of TIMPs (58). *In vitro* experiments confirmed that overexpression of miR-335 inhibits MMP-9 and collagen 1 promoter activity and protein expression (119). Moreover, recent studies showed that miR-21 promotes invasion by targeting MMP regulators (120, 121). Interestingly, pomegranate increased the level of anti-invasive miR-335 and decreased the expression of miR-21, which may represent antimetastatic targets of pomegranate (62). The expression of fibronectin as one of the other components of ECM decreased in breast cancer cells treated with pomegranate peel extract (53). Furthermore, hyaluronic acid (HA) is a nonsulfated glycosaminoglycan in ECM that regulates cell adhesion and migration. One of the HA receptors is hyaluronic acid-mediated motility receptor/CD168 (HAMMR), which is a nonintegral cell surface receptor (122). HA binding to HAMMR stimulates downstream signaling that activates RhoA-activated protein kinase and the MAPK/extracellular signal-regulated protein kinase (ERK) 1/2 pathway, which results in the expression of genes that are required for motility and invasion in various cancers (123, 124). The ﬁnding that pomegranate down-regulates HAMMR (62) suggests that the antimetastatic eﬀect of pomegranate may be partially due to inhibiting the HA signaling pathway. 

tenascin-C (TNC) is one of the other ECM glycoproteins that drives the progression of many types of human cancer (125). TNC is able to interact with several ECM proteins and many cell-surface receptors, which enables it to influence cell migration (126). There is evidence that miR-335 acts as a metastasis-suppressive miRNA in breast cancer by inhibiting type I collagen and TNC (127). Interestingly, pomegranate increased the expression of miR-335 and inhibited the expression of collagen I and TNC (62).

***Inflammatory and angiogenic molecules as targets of pomegranate***

It has been increasingly recognized that inflammatory components fire up cancer metastasis. Interestingly, the expression of some inflammatory chemokines was decreased with treatment of pomegranate seed oil in breast cancer cells (54). Moreover, pomegranate juice significantly reduced the level of secreted IL-6, IL-12p40, IL-1β, and RANTES (regulated on activation, normal T Cell expressed and secreted) in prostate cancer cells (128). Previous studies showed that overexpression of these inflammatory cytokines confers migratory and invasive properties to many tumor cell lines (129). Moreover, pomegranate extract suppressed the expression of critical mediators of inflammation markers, including those of inducible nitric oxide (iNOS), COX-2, prostaglandin E2 (PEG-2), and HSP90 (99, 130). 

Pomegranate extract has been found to down-regulate the expression of angiogenic factors such as vascular endothelial growth factor (VEGF). Moreover, previous studies showed that pomegranate seed oil and fermented pomegranate juice inhibited the expression of VEGF and increased expression of migration inhibitory factor (MIF) in breast cancer cells (56). It has been shown that pomegranate extract minimizes angiogenesis, tumor size, and metastasis by reducing the expression of VEGF (61).

***Perspective and limitation***

The overall purpose of this review is to briefly discuss the molecular targets of pomegranate with respect to cancer metastasis. Previous studies on pomegranate were mostly focused on fruit peel and juice. The antimetastatic activity of other tissues of pomegranate such as leaves, flower, seed, heartwood, and root should be studied in more detail. Moreover, as yet the antimetastatic effect of only a limited number of phytochemicals in pomegranate has been studied. Further *in vitro* assays and animal model studies should be performed to unravel the effects of other phytochemicals of pomegranate on the process of metastasis. Many phytochemicals in pomegranate are similar to other medicinal plants, so identification of underlying effect mechanisms of pomegranate will also promote our knowledge of molecular targets of other functional food plants. 

Additionally, more comprehensive studies should be performed on the expressional changes of metastasis related molecules following pomegranate use. A better understanding of the molecules regulated by pomegranate will undoubtedly enable its better clinical application.

## Conflicts of Interest

The authors declare that there are no conflicts of interest regarding the publication of this paper.
